# Nephrological Detrimental Impacts Resulting From Novel Immunotherapy Drugs Used in the Treatment of Cancer: A Systematic Review

**DOI:** 10.7759/cureus.54487

**Published:** 2024-02-19

**Authors:** Amisha Jaiswal, Kainaat Shergill, Kusalik Boppana, Naiela E Almansouri, Saloni Bakkannavar, Youmna Faheem, Tuheen Sankar Nath

**Affiliations:** 1 Internal Medicine, California Institute of Behavioral Neurosciences & Psychology, Fairfield, USA; 2 Surgery, California Institute of Behavioral Neurosciences & Psychology, Fairfield, USA; 3 Internal Medicine, University of Tripoli, Tripoli, LBY; 4 Pediatrics, California Institute of Behavioral Neurosciences & Psychology, Fairfield, USA; 5 Pediatrics, Ras Al Khaimah (RAK) Medical & Health Sciences University, Ras Al Khaimah, ARE; 6 Surgical Oncology, Tata Medical Centre, Kolkata, IND

**Keywords:** acute kidney injury, anticancer treatment, renal side effects, novel immunotherapy drugs, cancer treatment, immunotherapy, nephrological adverse effects

## Abstract

The most recent advancements in cancer therapy center on efficiently and conveniently enhancing a patient's natural immune system. Immune checkpoint inhibitors (ICIs) are antibodies that target cytotoxic thymus (T) lymphocyte antigen-4 (CTLA-4) and its receptor. They function by stimulating T-cell activity against malignancies. Immune-related adverse events (irAEs) are a distinct class of inflammatory side effects that are specific to a given organ. Antineoplastic medications can impact any part of the kidney, leading to the development of proteinuria, hypertension, electrolyte abnormalities, glomerulonephritis, and both acute and chronic interstitial nephritis. We reviewed the scientific literature regarding kidney problems that can arise from chemotherapy and immunotherapy for neoplasms, such as various cancers, melanoma, non-small cell lung cancer, and colorectal cancer. We discussed the pathophysiology, associated risk factors, management, and safety measures for patients experiencing acute renal injury after a new immunotherapy medication treatment. Antineoplastic drugs have the potential to damage the renal tubules, glomeruli, parenchyma, and blood vessels, among other kidney tissues. This can result in a broad spectrum of complications, spanning from a rise in serum creatinine levels without symptoms to the development of acute kidney injury (AKI). The research examined a range of risk factors associated with acute kidney injury (AKI). These factors encompassed age, gender, preexisting medical conditions (such as diabetes, hypertension, and chronic kidney disease), and the medications that patients were taking at the beginning of the study, which included non-steroidal anti-inflammatory drugs, renin-angiotensin system inhibitors, allopurinol, diuretics, corticosteroids, and proton pump inhibitors. The data suggests that patients who were receiving baseline treatment with proton pump inhibitors (PPIs) or corticosteroids had a higher risk of mortality. This study serves as an illustration of the effective management of acute kidney injury and proteinuria linked to novel immunotherapy drugs like pembrolizumab. The approach involved the use of corticosteroids tailored to the patient's condition. Furthermore, it references the recommendations outlined in the Common Terminology Criteria for Adverse Events (CTCAE). Prompt recognition and effective management of these side effects are essential to optimizing outcomes for patients undergoing immunotherapy. Our results were refined and focused by utilizing Medical Subject Headings (MeSH) keywords in our search strategy. The MeSH keywords used were "renal side effects" OR "immunotherapy" OR "cancer treatment." The studies reviewed encompassed a total of 48,529 participants among the 21 studies examined.

## Introduction and background

“Immunotherapy holds the promise of unleashing the body's own remarkable defense system to fight cancer." Dr. Suzanne Topalian. In 1891, Dr. William B. Coley used the first immunotherapy to save a patient with inoperable cancer [1]. The use of substances to strengthen and restore the immune system's capacity to fend off illness is known as immunotherapy. Immunotherapy seeks to regulate the immune system to eradicate cancer cells while preventing uncontrollably high levels of autoimmune inflammation, which can lead to immunotherapy's therapeutic limitations [2]. Due to their independent observations of significant tumor-shrinking after erysipelas infection, two German physicians, Fehleisen and Busch, are credited with initiating the field's first attempts to influence patients' immune systems for the treatment of cancer [3]. In transplantable mouse colon cancer and fibrosarcoma models, Allison JP et al. verified the theory that cytotoxic thymus (T)-lymphocyte-associated protein 4 (CTLA-4) inhibition could enhance the anti-tumor immune response [4]. Mice research showed that dendritic cells are essential in causing the development of regulatory T cells. Inventive methods used in immunotherapy that are currently being studied are bacterial-derived nucleotide immunostimulatory sequences or monophosphoryl lipid A, which amplify T helper cell reactions [5]. By either boosting the immune system's capacity to fight cancer or limiting tumor evasion mechanisms, active and passive immunotherapies have both been shown to be successful against a variety of cancers. Checkpoint inhibitors, lymphocyte-promoting cytokines, T cell receptor (TCR), T cells and other modified T cells, agonistic antibodies against co-stimulatory receptors, and cancer vaccines are different ways that immunotherapies are delivered [6]. Chimeric antigen receptor (CAR) therapy is an example of active immunotherapy that aims to teach the patient's immune system to recognize and fight cancer cells. On the other hand, passive immunotherapy, like checkpoint inhibitors, aims to dampen inhibitory T cells, thereby activating cancer-fighting T cells. There are two primary methods of employing immunotherapy: one is stimulating the natural defenses, which involves enhancing the innate capabilities of the immune system and prompting it to operate more vigorously or intelligently in identifying and attacking cancer cells, and the other is laboratory-produced substances; scientists can create substances in a laboratory that closely resemble components of the immune system. Immunotherapy medications are more recent, so there is less data regarding exposure's long-term effects. Chemokines have pro- and antitumorigenic effects and are crucial in determining the makeup of the tumor microenvironment since they are immune cell trafficking mediators. In this case, chemokines may serve as useful prognostic indicators of immunotherapy response [7]. Insights can be gained by carefully analyzing the immune cell profiles invading the tumor and the methods by which cancer might avoid the immune system, offering potential opportunities for the creation of novel treatment techniques [8]. The use of the immune system to fight cancer cells has its potential negative effects, just like other cancer treatment modalities like chemotherapy, radiation therapy, and surgery. The negative effects of immune checkpoint inhibitors (ICIs) are exclusively known as immune-related adverse events (irAEs) that can occur during treatment. Unfavorable circumstances, like a condition where the immune system is overactive and mistakenly harms healthy cells [9], are known. T-cell activity against malignancies is stimulated by immune checkpoint inhibitors (ICIs), which include antibodies that target the cytotoxic T lymphocyte antigen-4 (CTLA-4) as well as programmed cell death protein 1 (PD-1) and programmed cell death protein 1 ligand (PD-L1). However, this may lead to distinct immune-related adverse events, also referred to as inflammation-related side effects [10]. Early recognition and appropriate management of immune-related adverse events (irAEs) are paramount for ensuring patient safety and the effectiveness of treatment. Hence, it is crucial for healthcare providers working with cancer patients to possess a comprehensive understanding of these adverse events. IrAEs can affect various organs and systems within the body, with notable impacts on the kidney, skin, gastrointestinal tract, nervous system, and endocrine system. Although kidney problems following immune checkpoint inhibitors (ICIs) are believed to be relatively rare, they may be underreported. Given that early detection of kidney-related issues profoundly influences a patient's prognosis, having knowledge of potential symptoms and their management is an essential part of a physician's expertise [9]. This leads to certain important issues, like potential renal side effects associated with immunotherapy, including conditions such as immune-related nephritis and immune-related glomerulonephritis. The frequency of kidney-related adverse events in patients undergoing immunotherapy varies, but these side effects are generally considered uncommon. The likelihood of kidney-related side effects can differ among various types of immunotherapies. For instance, checkpoint inhibitors, a type of immunotherapy that targets certain proteins to enhance the immune response against cancer cells, have been associated with immune-related nephritis, although the occurrence of these side effects is relatively rare. The Intersect Fellowship Program for Computational Scientists and Immunologists was introduced by the American Association of Immunologists in 2018. Enhancing comprehension and communication between immunology researchers and computer scientists is the aim of the intersect fellowships [11]. Readers should bear in mind that the future of cancer immunotherapy is yet to unfold. In the next few decades, new immune checkpoint targets and combinations, tri-specific killer engagers, bifunctional checkpoint-inhibitory T cell engagers, chimeric antigen receptor (CAR) natural killer cell therapy, and inventive cancer vaccines are anticipated to be incorporated into clinical practice, alongside other still-developing methods [12]. The objective of this research was to investigate the occurrence, potential contributing factors, and underlying reasons for acute kidney injury (AKI) within a practical group of patients who received different types of immunotherapy treatment for carcinoma.

## Review

Methods

We rigorously followed the Preferred Reporting Items for Systematic Reviews and Meta-Analyses (PRISMA) guidelines to maintain the study's openness, thoroughness, and methodological rigor. The comprehensive review of pertinent literature was quite successful. By using the PRISMA methodology, it is made easier. To make certain that all pertinent studies were included, well-formulated and blended search terms were used. Only research directly related to the study was included according to clearly stated inclusion and exclusion criteria, and many questions were taken into account. The examination PRISMA protocol was followed, and papers that met the requirements were chosen after preliminary examinations of the titles, summaries, and full-text evaluations.

Database Search Protocol

The protocol for conducting a systematic review database search was meticulously designed to guarantee a thorough exploration of pertinent studies. To find pertinent material, three different databases were used. We refined and focused the search results using boolean operators and Medical Subject Headings (MeSH) keywords. The clever application of the boolean operators "AND" and "OR" allowed us to combine search phrases to cover a large number of resources while maintaining a high level of relevancy. When necessary, MeSH keywords were used to improve the search's accuracy. To ensure the best possible retrieval of relevant research, the search method was tailored to each database's syntax and structure.

The search strings used for composing the article are shown in Table 1. 

**Table 1:** Search strings

Database	Search String
PubMed	(“kidney side effects OR renal adverse effects OR nephrological side effects OR acute kidney injury OR ( "Acute Kidney Injury/drug therapy"[Majr] OR "Acute Kidney Injury/etiology"[Majr] OR "Acute Kidney Injury/mortality"[Majr] OR "Acute Kidney Injury/pathology"[Majr] OR "Acute Kidney Injury/physiopathology"[Majr] OR "Acute Kidney Injury/ therapy"[Majr] ) AND novel immunotherapy OR ( "Immunotherapy/adverse effects"[Majr] OR "Immunotherapy/mortality"[Majr] ) AND neoplasm treatment OR cancer treatment OR malignancy treatment OR tumor treatment OR ("Neoplasms/drug therapy"[Majr] OR "Neoplasms/ immunology"[Majr] OR "Neoplasms/therapy"[Majr])
Google Scholar	"renal side effects" OR "immunotherapy" OR "cancer treatment"
Medline	renal side effects of immunotherapy

The studies were screened based on the title of the studies, followed by a screening based on the abstract.

Inclusion Criteria

Included were the articles with human subjects and studies that had been released during the previous five years. This results in the extraction of data from the most recent research. Clinical trials and observational studies (cross-sectional, case-control, and cohort) were both acceptable for inclusion. These research plans included a variety of data sources for examination. To reduce publication bias, unpublished gray literature and published publications in peer-reviewed journals were included. English language studies were taken into consideration.

Exclusion Criteria

Since the focus was on human populations, investigations involving animals and in vitro experiments were not taken into consideration for inclusion. Research released beyond the previous five years was excluded. As a result, data from out-of-date studies is excluded. Due to limited data availability, conference abstracts, posters, and presentations without full-text publications were not included. To preserve coherence with the inclusion of peer-reviewed literature, unpublished dissertations were eliminated.

**Figure 1:**
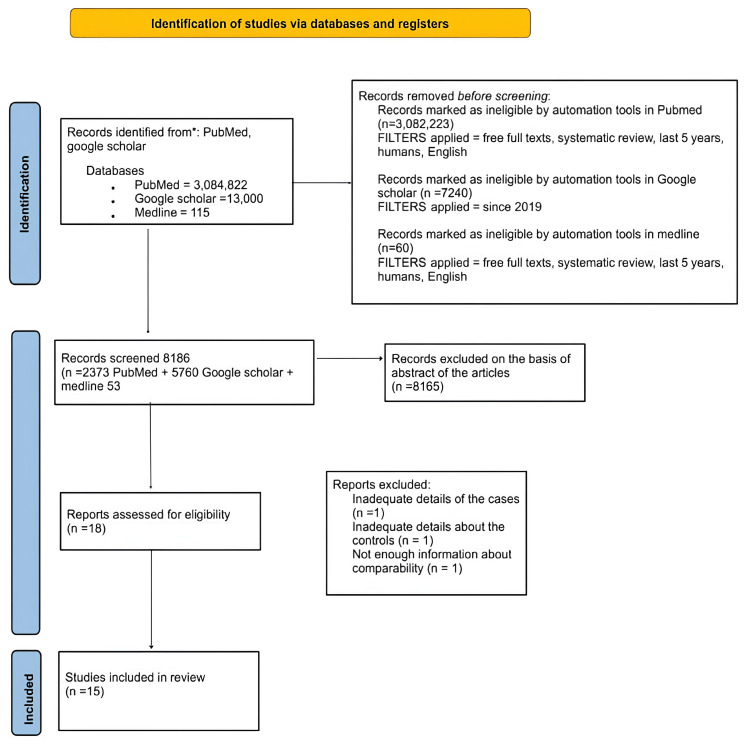
PRISMA diagram detailing the study identification and selection process PRISMA: Preferred reporting items for systematic reviews and meta-analysis

To ensure the study's thoroughness and methodological rigor, we strictly adhered to the Preferred Reporting Items for Systematic Reviews and Meta-Analyses (PRISMA) criteria, as illustrated in Figure 1.

Result

In the reviewed studies, a total of 48,529 patients were encompassed. Among the 15 studies analyzed, six were cohort studies, two were case-control studies, one was a systematic review, one was a narrative review, and five were case reports. The initial count of studies identified on PubMed was 3,084,822, Medline was 115, and on Google Scholar, it was 13,000. Applying filters such as free full texts, systematic reviews, articles from the last five years, humans, and English resulted in the exclusion of 3,082,223 records on PubMed and 60 results on Medline by automation tools. On Google Scholar, applying the filter for articles since 2019 led to 7,240 records marked as ineligible.

A total of 8,186 records were screened, including 2,373 articles from PubMed, 5,760 from Google Scholar, and 53 from Medline. Articles excluded based on the abstract were 8,165. For assessing eligibility, 18 articles were considered. The review ultimately included 15 studies, excluding three articles due to insufficient details regarding cases, controls, and comparability.

Table 2 depicts the concise synopsis of the articles that were cited in the paper.

**Table 2:** Succinct overview of the articles that were cited in the paper ICPi: immune checkpoint inhibitors; AKI: acute kidney injury; PPIs: proton pump inhibitors; RAASi: renin angiotensin aldosterone system inhibitors; C3GN: complement 3 glomerulonephritis; CPI: checkpoint inhibitors; AIN: acute interstitial nephritis; PD1i: programmed death 1 inhibitor; PD-L1: programmed death-ligand 1; TIN: tubulointerstitial nephritis; MN: membranous nephropathy; T-VEC: talimogene laherparepvec; IRAEs: immune-related adverse events; CRS: cytokine release syndrome; HLH: hemophagocytic lymphohistiocytosis; MAS: macrophage activation syndrome; CAR-T: chimeric antigen receptor.

Author	Year of publication	Number of patients	Type of Study	Subheading studied	Result/conclusion
Shruti Gupta [13]	2021	858	Case-control	Pathogenesis + associated risk factors	Individuals who experienced immune checkpoint inhibitor-associated acute kidney injury (ICPi-AKI) were more prone to having pre-existing renal impairment, using proton pump inhibitors (PPIs), and experiencing extrarenal immune-related adverse events (irAEs). About two-thirds of these patients exhibited a recovery of renal function after ICPi-AKI. The administration of corticosteroids was linked to enhanced renal recovery.
Claire Stein [14]	2021	239	Cohort study	Pathogenesis + associated risk factors	Acute kidney injury (AKI) frequently occurs in individuals undergoing anti-PD1 treatment for advanced melanoma, primarily manifesting as prerenal disease. The use of RAAS inhibitors (RAASi) tends to promote this condition. Renal immune-related adverse events (irAE) are infrequent in this context. Poor survival outcomes in this population are associated with the use of proton pump inhibitors (PPIs) and corticosteroids, while being overweight or obese appears to have a protective effect.
Meraz- MuñozA [15]	2020	308	Cohort study	associated risk factors	The majority of AKI patients in this group were mild to moderate in intensity, while severe cases responded well to systemic corticosteroids and a temporary cessation of ICPi. When patients who got AKI were given ICI again, there was no indication of damage. Re-challenge with ICPi may be an option for some patients, especially if their treatment choices are restricted. Other IRAE may be linked to AKI and should raise the possibility of ICPi nephrotoxicity in clinical practice.
Frank B. Cortazar [16]	2020	414	Case-control	Pathogenesis+ Treatment of AKI	This marks the initial occurrence of complement 3 glomerulonephritis (C3GN) with red blood cell-cast nephropathy resulting from immune checkpoint inhibitors (ICIs). The rarity of this case, linked to prolonged pembrolizumab use, reinforces the connection between ICIs and C3GN. Consequently, regular urine and renal function assessments are advisable for individuals undergoing pembrolizumab and other ICIs.
Zhi Yang [17]	2023	1	Case report	Treatment of AKI	Immunotherapy can lead to acute kidney injury, particularly in those with pre-existing kidney issues. Monitoring for renal complications, especially with ipilimumab and nivolumab, is crucial. Proton pump therapy may heighten this risk. Regular basic metabolic panel checks and prompt steroid use for immune checkpoint inhibitor-associated acute kidney injury (CPI-AKI) are advised.
Clarissa Casola [17]	2019	511	Cohort study	Pathogenesis	We suggest that employing immunohistochemistry with programmed death ligand 1 (PD-L1) could serve as a valuable tool to distinguish acute interstitial nephritis (AIN) associated with anti-PD-1 therapy from other forms of AIN.
Kevin Parza [18]	2021	1	Case report	Treatment of AKI	We encountered an unusual occurrence of pembrolizumab-triggered immune-related adverse events, manifested as Fanconi syndrome and type 1 renal tubular acidosis, emerging five months after the cessation of pembrolizumab. We strongly advise continued monitoring of tubular function, including assessing urine glucose or tubular injury markers, even after discontinuing pembrolizumab.
Brian Benes et. al. [19]	2023	1	Case report	Pathogenesis	Programmed death 1 inhibitors (PD1i) are known to induce acute kidney injury (AKI), often attributed to tubulointerstitial nephritis (TIN). The observed improvement upon discontinuing nivolumab and the subsequent onset of proteinuria after talimogene laherparepvec (T-VEC) administration suggest the potential for T-VEC-induced immune reactions.
Tella Sadighpour [20]	2021	6412	Narrative review	Pathogenesis	Immunotherapy effectiveness considers cellular characteristics, immune interactions, and patient tolerance. Oncologists and nephrologists should be aware of the elevated risk of AKI with immune checkpoint inhibitors (ICPIs). Early detection, potentially with a kidney biopsy, ensures proper management. ICPI-induced AIN has a distinct feature—a prolonged latency phase and milder AKI—distinguishing it from other drug-induced ATIN.
Bermejo [21]	2022	858	Cohort study	Pathogenesis	The spectrum of ICI use in kidney illness is broad; it includes its use in specific groups, such as dialysis and kidney transplant patients, as well as its relationship to immunotoxicity conditions such as AIN and glomerulonephritis. The renal adverse events (irAEs) linked to ICI treatment in patients with advanced cancer were emphasized in this review. Furthermore, we showed that randomized clinical trials with ICI involving patients with end-stage kidney disease and kidney transplant recipients are desperately needed.
Uppal NN [22]	2022	2556	Cohort study	Pathogenesis	The application of immunotherapy has been reported to treat a variety of electrolyte imbalances. Oncologists may be able to better manage the many unique electrolyte abnormalities linked with immunotherapy if they are identified early and diagnosed promptly.
Manohar S [23]	2021	11482	systematic review	Pathogenesis	Due to the extensive use of ICIs, novel irAEs have become part of clinical practice. About 2-4% of AKI cases are associated with ICI, and AIN accounts for more than 80% of these instances. An AIN diagnosis can be challenging without a kidney biopsy. The course of treatment involves stopping the ICI in patients with stage 2 or 3 AKI, identifying and eliminating any additional medications that are known to cause AIN, and utilizing corticosteroids. Because of the distinct side effects of CRS and HLH/MAS, chimeric antigen receptor therapy (CAR-T) is becoming increasingly important as an effective immunotherapy for cancer. It produces AKI, especially acute tubular damage, and prerenal azotemia.
Praveen Ratanasrimetha [ 24]	2022	1	Case report	Treatment of AKI	The first instance of probable late-onset ICI-associated MN is highlighted in this study, along with the growing difficulty in identifying renal irAEs.
Hayato Fujioka [ 25]	2021	1	Case report	Associated risk factors	This multicentre study reveals key information about risk factors, histopathological factors, and outcomes in patients experiencing immune checkpoint inhibitor-associated acute kidney injury.
Jagelia J [26]	2021	2945	Cohort study	Treatment of AKI	Renal difficulties may necessitate changing the therapy, lowering the pharmaceutical dosage, or permanently excluding a patient from a certain treatment plan. With chemotherapeutic medicines, it is considerably harder to predict potential adverse effects than it is with immunotherapeutic treatments because there is less information available on the pathomechanisms behind kidney damage in this mechanism.

Discussion

The process through which immunotherapy drugs cause kidney injury and the underlying mechanisms involved in this injury are discussed. Important questions about the renal outcomes, clinical characteristics, histological findings, risk factors, and overall survival of patients with ICPi AKI (immune checkpoint inhibitors associated with acute kidney injury) that remain unresolved are discussed.

Risk Factors and Associated Factors

The prevalence, risk factors, and underlying causes of acute kidney damage (AKI) in a cohort of patients receiving immunotherapy medications are clarified in this thorough research. Among the notable studies conducted in 2021, Shruthi Gupta's study involved 429 patients who were on immune checkpoint inhibitors discovered by oncologists from 40 significant academic cancer institutes. One hundred and twenty-one of these individuals (28.2%) had concomitant use of drugs for proton pump inhibitors (PPIs) and had acute tubulointerstitial nephritis (ATIN). The most widely used inhibitors are PPIs. A noteworthy finding indicates that ICIs were associated with an increased risk of ICPi-AKI, and other immunotherapy drugs were associated with recurrent ICPi-AKI: 16.5% of patients who had a second round of PPI treatment experienced extrarenal immune-related adverse events (irAEs). ICPi-AKI typically manifests around 16 weeks after initiating immunotherapy, with some instances occurring more than a year later. Kidney injury often became evident within three weeks after the last immunotherapy cycle and presented with varying levels of severity, encompassing stage 1, stage 2, and stage 3 AKI, with certain cases necessitating renal replacement therapy. An intriguing observation from the study was that the baseline estimated glomerular filtration rate (eGFR) no longer correlated with ICPi-AKI in instances of moderate-to-severe kidney injury, suggesting that reduced renal reserve might render individuals more susceptible to acute kidney injury (AKI). This finding underscored the significance of PPIs as a notable cause of drug-induced acute tubulointerstitial nephritis (ATIN), particularly in patients undergoing immunotherapy [13]. Another research effort conducted by Claire Stein in 2021 concentrated on 239 patients undergoing treatment for advanced melanoma with anti-programmed cell death (anti-PD1) immunotherapy, revealing an incidence of AKI [14]. The study explored various risk factors for AKI, including age, gender, comorbidities (e.g., diabetes, hypertension, and chronic kidney disease), and baseline prescribed medications such as non-steroidal anti-inflammatory drugs (NSAIDs), renin angiotensin aldosterone system (RAAS) inhibitors, allopurinol, diuretics, corticosteroids, and proton pump inhibitors (PPIs). Interestingly, patients who developed AKI were more likely to have pre-existing chronic kidney disease (CKD), receive RAAS inhibitors, and have previously been treated with ipilimumab before anti-PD1 therapy. Furthermore, they received more treatment cycles and higher cumulative doses of antiPD1 drugs. According to the number of lines of anti-melanoma medication, body mass index (BMI), and the initial prescription of PPIs and corticosteroids, the study revealed variances in overall survival. A greater mortality risk was associated with second-line anti-PD1 antibody therapy when compared to initial treatment. Curiously, patients with a BMI of 25-29.9 kilograms (kg) per meter square (m2) or under 30 kg/m2 showed a reduced prevalence. Compared to people with a normal BMI (18.5-24.9 kg/m2), mortality risk is increased. Initial administration of PPIs, or corticosteroids, was linked to a higher risk of death. Furthermore, a retrospective real-life cohort analysis of 1016 Massachusetts General Hospital patients who received immunotherapy, mostly anti-PD1, for a variety of malignancies (primarily melanoma) revealed a 17% incidence of AKI, with 3% probably attributed to immunological causes. In this cohort, co-prescribing PPIs was widespread, affecting 75% of participants. It is important to acknowledge that this study had limitations, including the fact that very few kidney biopsies were done, that not all patients had complete data, that a tiny percentage of patients were lost to follow-up, and that the cause of AKI could not be identified with sufficient certainty. AKI was associated with a history of cerebrovascular illness with an odds ratio (OR) of 9.2; a 95% confidence interval (CI) of 2.1 to 40.0; p<0.001; and a pre-existing diagnosis of hypertension (OR 4.3; a 95% CI of 1.8 to 6.1; p<0.001), according to Merza's study, which included 309 patients on ICP. Additionally, AKI was associated with the use of angiotensin-converting enzyme inhibitors (ACEi), corticosteroid therapy (OR 1.9; 95% CI 1.1 to 3.6; p=0.03), diuretics (OR 4.3; 95% CI 1.9 to 9.8; p<0.001) or 2.9; 95% CI 1.5 to 5.7; p = 0.002) [15], or angiotensin-receptor blockers (ARB). AKI was not associated with the ICPi agent, cancer type, baseline serum creatinine, or eGFR. The research led by Sruthi Gupta reveals that renal injury often manifests within three weeks following the conclusion of the immunotherapy cycle. The severity varied, spanning stages 1, 2, and 3 of acute kidney injury (AKI), with certain cases requiring renal replacement therapy. In contrast, the study at Massachusetts General Hospital lacked an adequate follow-up duration. Claire Stein's investigation identified several associated risk factors, including age, gender, comorbidities (e.g., diabetes, hypertension, and chronic kidney disease), and baseline prescribed medications. However, Sruthi Gupta's study and the Massachusetts General Hospital study primarily emphasized the concurrent use of PPI and corticosteroids.

Pathogenesis of Kidney Injury Caused by Novel Immunotherapy Drugs

**Figure 2:**
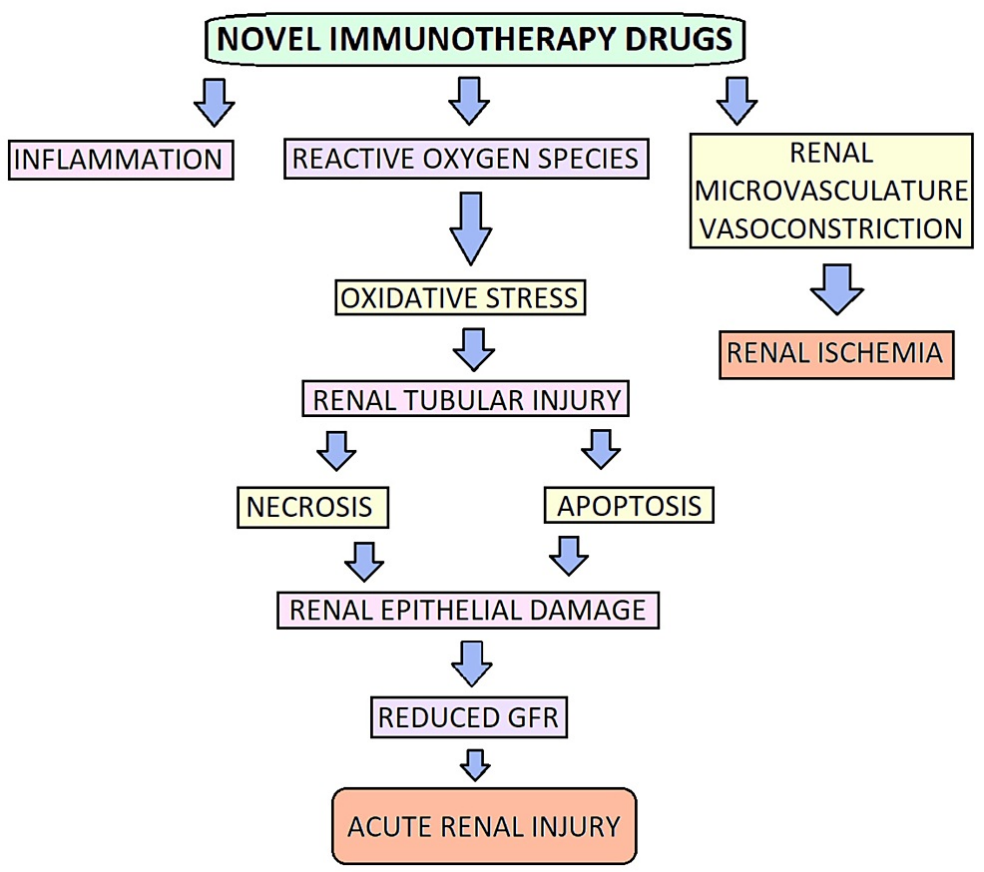
Representation illustrating the development process of acute kidney injury induced by emerging immunotherapy medications. GFR: Glomerular filtration rate

Acute kidney damage (AKI) is a prevalent side effect linked to several medical procedures, including cancer treatment methods. A reduction in kidney function can result from cancer treatments such as chemotherapy and immunotherapy, potentially increasing the risk of developing renal failure. Clinical signs such as proteinuria, hypertension, and electrolyte abnormalities are caused by nephron injury and stimulation. Acute kidney injury, acute and chronic interstitial nephritis, and glomerulonephritis can be caused by the adverse effects of immunotherapeutic drugs like ipilimumab, inhibitors of vascular endothelial growth factor (VEGF) (such as pembrolizumab, nivolumab, atezolizumab, bevacizumab, and ramucirumab), as well as several chemotherapeutic medications (such as capecitabine, irinotecan, gemcitabine, cisplatin, paclitaxel, carboplatin, docetaxel, vinorelbine, gefitinib, and erlotinib). This article emphasizes the pathogenesis, quantity, and sorts of renal problems that may result from particular treatment plans and when they might arise. A thorough evaluation of 48 clinical studies, including 11,482 participants, yielded a pooled estimated prevalence of AKI with PD-1 inhibitors of 2.2%. Acute interstitial nephritis (AIN) is the most commonly reported histological symptom of ICIs. In the first two case series of ICI-associated AIN, Cortazar and colleagues reported acute interstitial nephritis (AIN) in 12 of 13 patients, while Shirali and colleagues confirmed AIN in six patients. The majority of interstitial infiltrates are lymphocytes, with various amounts of plasma cells and eosinophils present as well. Granulomas are present in certain immune checkpoint inhibitor (ICI)-associated AIN patients as well [16]. In a study conducted by Zhi Yang [17], a 68-year-old man diagnosed with nonsmall cell lung cancer (NSCLC) who was undergoing pembrolizumab therapy developed significant medical issues after completing 19 cycles of treatment. The patient presented with noticeable symptoms, including gross hematuria (visible blood in the urine), severe lower-limb edema (swelling in the legs), and reduced urine output (oliguria). In response to these symptoms, a percutaneous kidney biopsy was performed to investigate the underlying cause. Upon microscopic examination of the kidney tissue, the biopsy revealed the presence of typical membranoproliferative glomerulonephritis (MPGN) in conjunction with acute tubulointerstitial nephritis. Red blood cell aggregates were present in the renal tubules, as evidenced by the red cell casts that were visible in the tubular cavities. Moderate complement component (C) 3 deposits (2+) were detected by immunofluorescence examination of the tissue in the mesangium both throughout the capillary loops and within the renal glomerulus. However, there was no significant staining observed for various markers, including immunoglobulin (Ig) G, IgA, IgM, kappa, lambda, C4, and C1q. Subendothelial electron-dense immune deposits in the kidney tissue were discovered by additional ultrastructural analysis. A diagnosis of C3 glomerulonephritis was made based on the patient's clinical symptoms, lab test outcomes, and pathological data from the kidney biopsy. It was determined that glomerulonephritis (C3GN) and red blood cell-cast nephropathy were present. AKI has been reported to be caused by programmed death-1 inhibitors (PD1i), most frequently resulting in tubulointerstitial nephritis. In research by Clarissa Castle [18], 15 patients who received anti-PD-1 therapy and experienced acute kidney damage (AKI) that necessitated a kidney biopsy were examined for morphological and immunohistochemical findings. Nine of these individuals had acute interstitial nephritis, according to the diagnosis, while six showed acute tubular necrosis (ATN) in addition to AKI. The research showed poor PD-1 staining in thymus (T) lymphocyte cells across all AIN and lupus nephritis patients. However, patients were found to have tubular epithelial cell membranes that stained positive for programmed death-ligand 1 (PD-L1). ATN, AIN brought on by other drugs, or people with lupus nephritis were observed. It is significant to highlight that one of the study's limitations was the fact that the majority of the biopsy samples only contained the renal cortex, which made it impossible to evaluate the staining pattern in the renal medulla. The expression of PD-1 and/or PD-L1 can be different in patients who develop renal problems while taking anti-PD-1 medication. Antigen-presenting cells, such as tumor cells, vascular endothelial cells, astrocytes, and pancreatic islet cells, are known to express PD-L1 cells. Although elevated PD-L1 expression has been seen in the renal tubules of patients with systemic disease, it was only noticed sometimes in tubules in normal kidneys with lupus erythematosus. ATN was the primary morphological finding among individuals without interstitial nephritis, and some biopsy samples revealed mild or focal interstitial inflammation that did not meet the criteria for an AIN diagnosis. Contrarily, all biopsy samples from patients receiving PD-1 inhibitor therapy showed localized PD-1 and PD-L1 staining of the cell membrane in patients with AIN. The staining of PD-1 was restricted to inflammatory cells; most of the time, this staining was weak and localized. On the other hand, PDL1 staining was often strong when seen in cells other than just inflammatory cells (like clusters of differentiation (CD) 3-positive, PD-L1-positive). T cells were seen concentrically along tubular epithelial cell membranes in regions of interstitial inflammation. The tubules with positive staining were significant. Patients taking antiPD-1 medication and those with AIN not linked with PD-1 showed a significant variation in the pattern of PD-L1 staining. In a different case study by Kevin Parza [19], a 73-year-old man with a complicated medical history, including metastatic uveal melanoma with liver metastases, stage 3A chronic renal disease, hypertension, type 2 diabetes, and hyperlipidemia, received his first dose of ipilimumab and underwent a liver biopsy three weeks before the diagnosis of acute tubulointerstitial nephritis (ATIN). The patient was immediately started on 60 mg of oral prednisone everyday treatment because there was a suspicion of drug-induced nephropathy. He underwent hemodialysis six times during his one-week inpatient stay. His estimated glomerular filtration rate increased, and his creatinine levels subsided. The eGFR (estimated glomerular filtration rate) increased. He was instructed to finish the steroid taper and stop using immunotherapy. Brian Benes et al.'s study [20] presented a case involving a 72-year-old man who had been undergoing adjuvant nivolumab treatment for melanoma and subsequently developed acute kidney injury (AKI). A kidney biopsy conducted in this case revealed distinctive pathological features. The biopsy findings indicated the presence of mesangial and focal endocapillary proliferative glomerulonephritis. When subjected to immunofluorescence analysis (IF), the kidney tissue exhibited positive staining for complement (C3) and trace amounts of complement 1Q (C1Q). Furthermore, electron microscopy revealed the existence of electron-dense mesangial deposits in the renal tissue. This case highlights the complex and varied presentations of kidney injury associated with immunotherapy and underscores the importance of tailored treatment approaches based on the specific underlying immunological mechanisms. Pembrolizumab, an immune checkpoint inhibitor, can occasionally lead to acute tubulointerstitial nephritis as an immune-related adverse event. Immune checkpoint inhibitors like pembrolizumab induce an inflammatory immune response involving cytotoxic effector CD 8-positive T cells, which can escape the tumor microenvironment. However, these immune-related adverse events are not limited to the tumor site and can affect other organs as well. In a retrospective study conducted by Tella Sadighpour [21], renal biopsies were performed on 16 out of 6412 cancer patients who had undergone immune checkpoint inhibitor (ICPI) therapy. The study included individuals with an average age of 64 years and spanned 10 years following ICPI treatment. The researchers observed that the manifestations of acute kidney injury (AKI) became noticeable between the 6th and 56th weeks after the initiation of ICPI therapy, with a median onset at 14 weeks. Acute tubulointerstitial nephritis (ATIN), which was detected in 14 of the 16 renal biopsies, was the most frequent morphologic result. The study also found that these patients had several glomerular diseases, including pauci-immune glomerulonephritis, C3 glomerulonephritis, immune complex-mediated glomerulonephritis, IgA nephropathy, and glomerulopathy with membranes. In conclusion, one of the significant limitations of immune checkpoint inhibitors (ICPIs) in clinical use is their potential for nephrotoxicity. This nephrotoxicity can manifest in various forms, which are all separate adverse effects connected to infusions and include glomerular disorders, acute interstitial nephritis (AIN), and acute tubular necrosis (ATN). Immune cell infiltration is the fundamental pathophysiology of ICPI-induced nephrotoxicity. Plasma cells, eosinophils, and T lymphocytes enter the renal tissue, causing interstitial injury. Acute tubulointerstitial nephritis (ATIN) is the end outcome of inflammation and edema. The disruption of self-tolerance by ICPIs results in an adverse reaction, which may be the cause of this nephrotoxic impact. In numerous organs, including the kidneys, the autoimmune response is directed against particular self-antigens. Although the precise nature of these antigens is still unknown, the greater incidence of ATIN found in kidney biopsies is proof that renal tubular cells are involved. Bermejo's study mentions that tumor necrosis factor-alpha (TNF-alpha) and interleukin-9 (IL-9) levels in the urine were higher in this group when compared to other kidney pathologies that were biopsied, such as acute tubular necrosis, diabetic nephropathy, or glomerulopathies [22]. Moledina's prospective study which involved 218 patients, of whom 15% were diagnosed with AIN. A recent review of the Food and Drug Administration Adverse Event Reporting System (FAERS) on ICI from 2011 to 2021 revealed 2556 recorded occurrences of electrolyte abnormalities, with hyponatremia being the most often reported electrolyte abnormality [23]. Kitchlu et al. conducted a systematic analysis and discovered 45 case reports of biopsy-confirmed ICI-associated glomerular illness [24], with pauci-immune glomerulonephritis being the most prevalent kind. Clarissa Cassol's study showed limited PD-1 staining in T cells for acute interstitial nephritis (AIN) and lupus nephritis patients, while Tella Sadighpour's study identified acute tubulointerstitial nephritis (ATIN) in 14 out of 16 renal biopsies as the most common morphological result. Whereas Kitchlu's study highlights the electrolyte abnormalities associated with ICIs. Zhi Yang's and Clarissa's study depicts the various findings of the kidney biopsies of the patients involved in the study, representing the role of different complements and immunoglobulins involved. In summary, the nephrotoxic potential of ICPIs highlights the need for vigilance and close monitoring of renal function in patients receiving these immunotherapies, as early detection and intervention are crucial for managing and mitigating renal complications. Further research is needed to gain a deeper understanding of the precise mechanisms underlying ICPI-induced nephrotoxicity and to develop more targeted therapeutic approaches. Figure 2 illustrates the development process of acute kidney injury induced by emerging immunotherapy medications.

Treatment and Prognosis

The studies discussed offer valuable insights into the treatment and outlook for acute kidney injury (AKI) associated with immunotherapy. The case of an 80-year-old patient with metastatic lung adenocarcinoma who experienced acute renal injury and significant proteinuria following pembrolizumab is described in Marco Bonilla's paper [25]. The patient benefited from corticosteroid treatment and finished a total of six months of therapy. Notably, the urinary protein/creatinine ratio showed remarkable improvement, decreasing to 1.2 grams (g) within two months of treatment and further reducing to 0.2 g at the six-month post-treatment evaluation. Additionally, the patient's serum creatinine levels returned to their baseline, which is 1.19 milligrams (mg) per deciliter (dl). Following the completion of corticosteroid treatment, the patient remained free of kidney disease recurrence, and there was no disease progression observed in his lung cancer at the six-month follow-up. This case illustrates the successful management of acute kidney injury and proteinuria associated with pembrolizumab through a tailored treatment approach involving corticosteroids. It also highlights the importance of interdisciplinary collaboration in making informed decisions for patients with complex medical conditions. The limitation of this study is a smaller sample size as compared to other studies. In another study by Hayato Fujioka, a 71-year-old woman with recurrent mandibular gingival cancer received combination chemotherapy with pembrolizumab, cisplatin, and fluorouracil, followed by pembrolizumab monotherapy. When this treatment failed to stop the cancer's progression, secondary chemotherapy with cetuximab and paclitaxel was initiated. Following the development of various symptoms, a renal biopsy was done. The biopsy of this patient revealed inflammatory changes, including tubulitis in the proximal tubules. Lymphocytes were observed in the subcapsular interstitium, with mainly CD 20-positive cells [26], while the peritubular and interstitial areas of the renal cortex contained CD3-positive cells, with a distribution of both CD4-positive and CD8-positive cells. Hydrogen-adenosine triphosphatase expression was reduced in damaged tubular epithelial cells. Immunofluorescence staining showed no deposition of immunoglobulins or complements in the glomeruli or tubular basement membrane, confirming the diagnosis of acute tubulointerstitial nephritis, which was relieved by the use of corticosteroids. It's important to note that this study is a case report, and as such, the sample size is limited, and the follow-up period is not extensive. According to Jagieła's study, steroid medication is advised until grade 1 creatinine readings are attained. For grade 2, the duration of methylprednisolone weaning should be 2 to 4 weeks, and for grades 3-4 ≥4 weeks. Maintaining adequate hydration is a frequently employed and advised strategy to avoid nephrotoxicity [27]. The management of ICI-associated AKI is discussed in Marco Bonilla's study, along with the course and use of corticosteroids in the same. It also mentions that the complex nature of some medical conditions necessitates collaboration among various specialists to ensure optimal decision-making for patients. However, Jagieła's study provides valuable insights into corticosteroid use for kidney injury, including treatment adjustments based on injury severity, the importance of follow-up, and the key role of hydration in preventing kidney damage. Thus, research on the impact of various drugs, such as immunotherapeutics and chemotherapeutics, on renal function is crucial. Creating uniform guidelines is also essential to promote collaboration between nephrologists and oncologists before and after cancer patient treatment.

Limitations of the study

Several limitations of this study should be acknowledged. First, the inclusion of several case-control studies with relatively small sample sizes might impact the generalizability of the findings. Additionally, the relatively short follow-up duration for the patients in these studies could potentially limit our understanding of long-term outcomes. Another limitation is the absence of a control group in many case reports, making it challenging to attribute observed outcomes solely to the treatment in question, as other factors may be at play. Furthermore, this study focused exclusively on articles published in the English language, potentially excluding relevant research in other languages. Lastly, the selection of papers published only within the last five years may have restricted the historical context of the analysis. Restricted access to free full-text articles may result in the potential omission of valuable research papers.

## Conclusions

In conclusion, as the utilization of immune checkpoint inhibitors (ICIs) continues to expand for the treatment of various malignancies, it becomes increasingly imperative to place a heightened focus on the potential side effects associated with these therapies, particularly those related to renal toxicity. The intricate interplay between the immune system and these inhibitors underscores the necessity for a comprehensive understanding of the possible adverse effects. Recognizing and addressing renal complications early in the course of immunotherapy are vital steps in ensuring the best possible outcomes for patients undergoing these treatments. This emphasizes the importance of ongoing research, vigilant monitoring, and the development of effective strategies for the early detection and management of ICI-related renal side effects, ultimately contributing to the enhancement of the overall safety and efficacy of immunotherapeutic interventions.
